# Treatment of Uterine Displacements by Genu-Pectoral Posture and Pneumatic Pressure

**Published:** 1875-11

**Authors:** T. S. Hopkins

**Affiliations:** Thomasville, Ga.


ATLANTA

MEDICAL AND SURGICAL JOURNAL.

VOL. XIII.] NOVEMBER—1875. [NO. 8.

### Original Communications.

### TREATMENT OF UTERINE DISPLACEMENTS BY GENU-PECTORAL POSTURE AND PNEUMATIC PRESSURE.

BY T. S. HOPKINS, M.D., OF THOMASVILLE, GA.

*Read before the South Georgia Medical Society at Bainbridge, Ga., Sept.* 26, 1875.

At the meeting of the Medical Association of Georgia, at Savannah, in April last, Dr. H. F. Campbell, of Augusta, presented the synopsis of a very able report, (since published in the ATLANTA MEDICAL AND SURGICAL JOURNAL,) on the treatment of Uterine Displacements by Knee-breast Posture and Pneumatic Self-repositor, the rationale of which was so clearly and philosophically demonstrated by the pneumatic pump which he used that I determined to overlook no opportunity for testing it. Since the time mentioned, I have fairly tested the treatment in six cases, and it affords me pleasure to state that it has been an entire success in every case.

Most of us have time and again resorted to the knee-breast-posture in reducing uterine displacements, and those of us who have must be aware that, though the posture aided us in replacing the organ, as a curative measure *per-se*, it was a failure, the displacement recurring on the woman's resuming the erect posture. This, doubtless, suggested to the philosophical mind of Dr. Campbell the "Pneumatic Self-repositor," the *sine qua non* by which he has conferred a blessing upon woman in enabling her to manage her own affairs without the assistance of the doctor. It is unnecessary, and would be surperfluous, for me to enter into any discussion of the rationale of Dr. Campbell's treatment, as it has been published to the world, and doubtless read by all present. I speak of it as Dr. Campbell's treatment for the reason that, though the posture part is as old as the hills, the pneumatic self-repositor is his invention, and is really the *sine qua non* in the treatment.

Since the first of May last I have treated six cases of uterine displacement by this method—four of which were prolapsus, one procidentia, and the other ante-version in early pregnancy, attended with constant nausea and vomiting. I will give but one case of Prolapsus as typical: Ellen, aged thirty-five years, mother of five children, had been an invalid for several years. During the past year she had been most of the time confined to her house, and much of the time to her bed; complained continually of pains in head, back, side, and almost everywhere else, with difficulty in passing water, and frequent attacks of hysteria; her husband, several times, reporting her in a dying condition, with cold extremities, palpitation of the heart and inability to move or speak. She had passed under the batteries of quackery for heart disease, dyspepsia, consumption, and various other things. When I saw her she had recently received a broadside from a cancer battery. In spite of the treatment, she continued to live. Campbell's treatment was applied, and from that day to the present, a period of four months, she has never had a "turn," the nervous symptoms have vanished and she is now attending to her household and out-door duties, "feeling like another person" —has not felt so well for years. What has produced this wonderful change in the condition of this poor woman, who, for years, had been a miserable invalid, living in continued fear of impending death, a burden to herself and her family? The problem is explained in a few words: This woman was laboring under *Prolapsus Uteri*, the organ was restored to its normal position by Campbell's treatment, and her protean ailments were gone. The treatment has been equally prompt and successful in all the other cases.

Procidentia—Emily, aged twenty-five years, the mother of one child; sought advice from me for procidentia uteri on the 4th of August, 1875. Procidentia had occurred soon after the birth of her child, more than a year since. In consequence of her inability to discharge the duties of nurse, she had been discharged from service. She was furnished with a repositor and instructed how to use it. She went home, and that night replaced the procidented uterus as she had been accustomed to do and commenced the treatment. On Sunday last, the 19th inst., I met Emily at a friend's house, so much changed that I did not recognize her. She informed me that, since the first application of the treatment, which she still continues, the womb has never "come down," and the back aches are gone. She urinates without difficulty, and is feeling well.

Since writing the above, Emily has called to see me. She is to all appearances well, and is again employed as a nurse.

The last case—that of ante-version—is a most interesting and instructive one, affording us the hope of relief in those distressing cases of nausea and vomiting in the early months of pregnancy, defying the best directed treatment heretofore known, and involving, occasionally, not only the loss of the child but the life of the mother. This case was a lady of our town, who had been confined to her house some six or eight weeks, it was said, with constant vomiting of almost everything taken into the stomach. She was said to be in the third month of her fifth pregnancy. I was called in consultation to the case on Friday last, 17th of the present month. I was then informed that her stomach had retained nothing in the way of nourishment for the past two weeks. During that time she had been nourished entirely by enemas. Her whole appearance indicated the truthfulness of the information given. I suggested the immediate and free use of diffusible stimulants. They were given and very soon thrown up. A vaginal examination revealing an ante-version, upon the theory of Mr. Hewitt that the nausea and vomiting of early pregnancy are consequent upon uterine displacements, it was determined, at my suggestion, to test the effect of genu-pectoral-posture and Campbell's Pneumatic Self-repositor. The patient was too much exhausted to assume the position without assistance. The repositor was inserted, and the position continued not exceeding ten minutes.

From that time to the time of her death, which occurred at 11 o'clock on the night of the 19th, the stomach retained, without the least nausea, everything in the way of nourishment and stimulus which was given, and it was given freely as the only hope. The treatment was applied on the 17th; on the morning of the 18th a vaginal examination proved the womb in normal position. *Query.*—Does not the result in this case warrant the belief that if the treatment had been applied earlier in the case, and before starvation had done its work, the life of the patient might have been saved? The replacement of the uterus in these cases is readily accounted for by pneumatic pressure, as explained by Dr. Campbell. But it does more than this: examination of the previously relaxed vagina, after several insertions of the repositor, will satisfy any one that the air admitted thereby exerts a powerful contractile influence on the tissues of that canal, bringing the walls in opposition and making a rest for the uterus, thereby relieving the suspensary ligament of the tension and consequent relaxation to which they have been subjected by the weight of that organ, and enabling them to recover their lost tone and functions. In this way the treatment is curative.

As I was unable to procure Dr. Campbell's repositor to use in the above cases, I used a tin tube manufactured for me by Mr. B. F. Fudge, a tinner. Though less comely than the glass repositor, they proved equally efficient. A repositor can be found in almost every house in this section of country. Wherever there is a coffee pot you have one by knocking off the spout and using it as such. In the absence of both tin and glass, I would make necessity the mother of invention, and use as a substitute a section of the common reed cane, which grows in any of your swamps and low grounds, having rounded off the edges. Indebted to Dr. Campbell solely for my success in the treatment of the cases here reported, it becomes my duty, whilst it affords me pleasure to publicly acknowledge the same, and return him my sincere thanks for the valuable service rendered.

Hoping, gentlemen, that other members of the society will proceed to test the merits of the treatment, and report the results, I respectfully submit this report.

